# Medical and Philosophical Intelligence

**Published:** 1813-05

**Authors:** 


### MEDICAL AND PHILOSOPHICAL INTELLIGENCE.

ROYAL SOCIETY.—On Thursday, January 28th, a paper by Dr. Brewster was read, *On some New Properties of Light*. This paper contained a kind of abstract of the author's numerous and interesting experiments on light. It was divided into four parts. 1. On a new property of light discovered in the agate. In a plate of transparent agate about $\frac{1}{15}$th of an inch thick, cut in a direction perpendicular to the laminæ, besides the colourless image of the object seen through this substance, there was another image strongly coloured. On viewing this coloured image through a prism of iceland crystal, it was found to possess the properties of doubly refracted light, disappearing at the quadrants when the prism was turned round. The colourless image possesses the same property. When a ray of light passes obliquely through a thin plate of agate, if it be received upon another plate of agate cut parallel to its coats, it easily passes through; but if the plate be cut in a direction perpendicular to the coats, it is totally reflected. Carnelian and chalcedony, which are kindred substances, possess analogous properties. Besides the two images above-mentioned, there is a nebulous appearance always visible in agate, probably occasioned by its want of complete transparency. This nebulous appearance Dr. Brewster is at present examining, hoping that it will throw additional light upon this curious but intricate subject. 2. *On the Double Refraction of Chromate of Lead.* Dr. Brewster found that the double refrangibility of chromate of lead is nearly three times as great as that of Iceland crystal. 3. *Power of different Bodies to refract Light.* Since Newton's time it has been conceived that the diamond possesses this power in the highest degree; but Dr. Brewster has found that both chromate of lead, and realgar, exceed it considerably in this respect. 4. *Double Dispersive Power of Bodies.* Dr. Brewster has found that bodies possess two different dispersions, one considerably greater than the other. This he discovered at first, by comparing his own measurement of the dispersive power of a body with that of Dr. Wollaston,

On Thursday, the 4th of February, a paper by Sir Charles Blagden was read, as an appendix to Mr. Ware's observations on shortsightedness. Sir Charles's own experience confirmed Mr. Ware's conclusions. When a boy of four years he was not short-sighted; but he was so, in a slight degree, at nine. He did not use glasses till 30. At first number 2 answered; then he required number 3; and at present the glass which he uses is number 5. He thinks that his shortsightedness has been encreased by the use of glasses, and considers it as probable that if he had not employed glasses it would have gone off altogether.

At the same meeting was read an account of the late eruption of the volcano called the Suffriere, in the island of St. Vincent, by Mr. Hamilton, of Nevis, in the West Indies. This mountain is the highest, and farthest north, of a chain of mountains which runs through the island. Its height is 3000 feet. It had been famous for its eruptions in former times; but none have occurred since 1718. The crater on the the top was a mile in diameter, of the form of an inverted cone, and about 800 feet deep. In the middle of it was a conical hill, covered with wood except the top. The mountain was covered with wood. It got its name from the general quantity of sulphur upon it. Through some of these sulphureous deposites a white smoke was always seen to issue. Some smoke also issued from the little conical hill at the bottom of the crater. The great number of earthquakes, no fewer than 200, during the course of 1811, announced that the entrails of the mountain were in a state of great commotion; but it continued to be visited by the curious; and even so late as the 27th of April, a party of gentlemen went to it, and spent several hours in examining the crater. On the 28th the eruption began by a prodigious shower of ashes, which continued to be thrown up all day, and which darkened the air, and loaded the wings of the birds to such a degree as to bring them to the ground. On the 30th, flames were first seen to issue out, accompanied by a vast column of smoke, and frequent thunder and lightning. On the 1st of May a prodigious shower of stones fell all over the island for about an hour, killed and wounded many negroes, and did prodigious damage. Two eruptions of lava took place: one ran down the north side of the hill, and reached the sea, distant about four miles, in four hours; the other ran east, choked up a considerable river, and did infinite damage. Much of the wood was destroyed, and several estates were so much covered with stones and ashes as to render it almost impossible to clear them. The ashes fell upon a ship at sea, more than 200 miles distant from the island.—

On Thursday, the 11th February, Mr. Hamilton's account of the Suffriere, in St. Vincent, was concluded.

—Some time after, a prodigious eruption of mud took place. It was so immense as to fill up all hollows and precipices, and has made the whole country so smooth that a coach and six might now drive up the hill. It was attended with a violent earthquake. Mr. Hamilton accounts for these phenomena, by supposing that the rivers whose courses were blocked up made their way into the burning mountain, and occasioned both the eruption and the earthquake. The inhabitants of Nevis were informed of the eruption of the Suffriere by loud and regular explosions, so like an engagement at sea that several of the vessels were deceived by it; and the Commodore of a convoy, conceiving that privateers were cutting off his sternmost ships, gave chase to the supposed enemy. Vessels 100 miles east from Barbadoes had their decks covered with sand, while vessels much nearer St. Vincent escaped without any such appearance. This Mr. Hamilton thought at first could only be accounted for by some eruption out of the sea to windward of the ships, or in some part of South America; but he seems afterwards to have altered his opinion.

On Thursday, the 18th February, a paper was read by Sir Everard Home, Bart. *on the Narwal,* proving that the tusk is peculiar to the male. It has been believed by naturalists that the narwal has two tusks, and that one of them is frequently broken off. A skull with two tusks was exhibited in the Leverian Museum. When that museum was exposed to sale, this preparation was examined, and it was found that one of the tusks had been artificially fixed on. The tusk is is upon the left side of the skull. On the right side there is the mark of a milk tusk which had been shed. In a conversation with Mr. Scoresby, jun. of Whitby, that gentleman told Sir Everard that sculls of the narwal were often found without tusks, and that it was a general opinion among the whale fishers that the tusk was peculiar to the male. Sir Everard earnestly requested Mr. Scoresby to procure him such a scull, which he promised to do. He has lately sent him a skull without any tusk, and assured him that it was taken from a female narwal; so that the fact of the tusk being peculiar to the male is made out beyond doubt.

A paper by Dr. Wollaston was also read, showing *A Method of drawing very fine Wire.* Muschenbroek mentions that an artist of Nuremberg drew gold wire so fine that 500 inches of it only weighed one grain; but he says nothing about the method. It may be done thus: take a wire of fine silver, of a moderate diameter, and drill a hole through its centre one-tenth of the diameter of the whole. Fill this hole exactly with a wire of fine gold. Draw out the silver wire, say to the fineness of $\frac{1}{300}$th of an inch. It is obvious that the gold wire will now be only $\frac{1}{3000}$ of an inch. Put the wire into warm nitric acid. The silver wtll be dissolved, and the gold wire will remain. It being difficult to drill a hole in fine silver, on account of its toughness, Dr. Wollaston tried platinum, and found it to answer. He made a mold for a silver wire, fixed in its centre a fine platinum wire, and then filled the mold with melted silver. The wire was now drawn out, and the silver dissolved by aqua fortis. By this means he easily obtained wires of the diameters of $\frac{1}{4000}$ and $\frac{1}{1000}$th of an inch, which is fine enough for every useful purpose. To make use of such wires, the best way is to take the requisite length before the silver is dissolved off, to give it the shape of an U, make a hook at each extremity, suspend it by a gold wire, dip it into the aqua fortis, and leave a portion of the silver at the ends, which will make it much more manageable. There seems to be no limit to this method, except the ductility of the metals. Dr. Wollaston obtained platinum wire as fine as $\frac{1}{30000}$th of an inch; but it was impossible to obtain it thus fine of any length.

---

*Linnæan Society.*—The Society met on the 19th of January. The paper read consisted of descriptions (accompanied by figures) communicated (from the executors of the late Mr. Anderson, of St. Vincent) by A. B. Lambert, esq. V.P.L.S. The first, a new species of psidium, (P. polycarpon,) ripened fruit last summer in Mr. Lambert's stove. It is a low shrub, with decumbent branches, bearing a round yellow fruit, the size of a large cherry, and of a flavor esteemed superior to that of the common guava. It is a native of the Savannahs of the Island of Trinidad, whence it was introduced into the botanic garden at St. Vincent's in 1792.—The second, a species of brownea, supposed by Mr. Anderson to be new, and in fact first discovered by him in Trinidad in 1786; but since described and figured by Jacquin under the name of brownea capitilla. It is a small tree, with large clusters of bright scarlet flowers, which were unknown to Jacquin.

On the 2d of February, a description of a plant found in India by Col.

Col. Hardwicke was read, which he conceived to constitute a new genus. It was found in the Mysore, and transported by the colonel to his garden, where it grew luxuriantly. It belongs to the natural order of *Malvaceæ*. Its greatest length was about 12 feet, and its stem was about 3 inches diameter. Hence it was too weak to resist violent winds when removed from the forest. It belongs to the class and order *Monodelphia Polyandria*. Its essential characters are, a double calyx, the outer of three leaves, the inner of five. The corolla has five petals. The seed-vessel is divided into ten cells, containing each about ten seeds in two rows. Colonel Hardwicke, if it should turn out to be a new genus, proposes to name it after Lord Valentia, *Annesleyana*.

A description of seven new species of shells by Mr. Brooks, described from his own collection, was also read. The first a chama, which was figured by Gualtieri, but not hitherto considered as a new species. He calls it chama Gualtieri. Six species of halyotis. Mr. Brooks was not acquainted with the place where any of these were found, except one, and that was the Pacific Ocean. It is very much to be lamented that the habitation of shells, and the animals that inhabit them, are not more attended to by collectors. It is the only way to perfect this difficult department of natural history.

At the meeting which took place on the 16th of February, the following communications were read :—

A letter from Mrs. Taylor, with specimens of a gelatinous plant, conceived to be a new species of lichen, which grew two successive years near her house in Devonshire.

A letter from Mr. Hoy, at Gordon Castle, Scotland, giving an account of a fish cast ashore near Gordon Castle, the trichiurus lepturus, not hitherto conceived to exist upon the British coast. The specimen, without the head, was about 12½ feet long.

A letter from Dr. Smith, President of the Linnæan Society, giving an account of the astragalus campestris, a new British plant, lately discovered on a rock in Angus-shire, by Mr. George Don. It is a splendid plant, and quite alpine. It was described by Haller. In the Flora Danica it is figured under the name of astragalus uralensis. Willdenow describes it under the name of astragalus sordidus.

An account by Mr. Kirby of a new species of insect, from an imperfect specimen sent him by Mr. Sowerby.

An account of a tree observed in the West Indies by Mr. Push, belonging to Jussieu's order of *Euphorbia*, and to the *Monoecia Monodelphia* of Linnæus. It constitutes a new genus. Mr. Push names it, in honor of Dr. Hosack, of New York, Hosackia laurina.

---

*Wernerian Natural History Society.*—The following papers have lately been read at the Wernerian Natural History Society :

I. A mineralogical description of the Ochil hills ; by Charles Mackenzie, esq. The first part of this valuable paper contained a geographical delineation of that beautiful and interesting range of hills. The second part contained a full and accurate account of all the rocks of which it is composed, beginning with the lowest or oldest, and ending with the uppermost or newest. The following are are the rocks described by Mr. Mackenzie, arranged according to their relative antiquity, the first-mentioned being the oldest, the last the newest: 1. Red sandstone. 2. Amygdaloid. 3. Grey sandstone. 4. Limestone. 5. Slate clay. 6. Claystone. 7. Tuff. 8. Basaltic clinkstone. 9. Greenstone. 10. Claystone porphyry. 11. Compact felspar. The third part contained a short account of the different veins that traverse the strata of the district; these are calcareous spar, streatite, heavy spar, iron, cobalt, silver, copper, and lead.

II. A paper by Professor Jameson, on the distribution of carbon throughout the mineral kingdom.

III. A memoir by Professor Jameson, on universal and partial or local formations.

IV. A journal kept in the Greenland seas, during the summer of 1812, by Mr. Scoresby, jun. of Whitby; which contains much curious information: by the same gentleman, a short but interesting notice respecting the Polar Bear.

V. An account of the old silver mines in Linlithgowshire, by Mr. Fleming: also a description of a great bed of sea shells, observed a considerable height above the high-water mark of the Frith of Forth; and an account of the Sorex fodiens, found by M. Fleming, in Fifeshire.

VI. Dr. Yule, in continuation of his physiological observations on mono-cotyledonous plants, read a memoir on the characters of the *Triticeæ* and *Hordaceæ*, as distinct natural orders. It is singular, he observed, that an assemblage of plants, occupying by far the greatest portion of the surface of the habitable globe, existing under every extreme of temperature, from between the tropics to the vicinity of the poles, should have remained so imperfectly known, as to be still considered as *one* natural order: for, since the accurate description and delineation of upwards of 400 species, by Scheuchzer, enabled Linnæus to characterise the genera then known, and the addition of others by Konig and Thunberg, with the numerous species described by Leers, Swartz, Schreber, and Brown, no subdivision of these plants has been effected on the principles of the natural method, even that of Jussieu being merely artificial. The present, therefore, is the first attempt to arrange a part of this extensive series on the principle of natural affinity.

---

*Vegetables found in some species of Minerals.*—Professor Blumenbach, of Gottingen, in a letter to Von Moll, says, that though he had hitherto disbelieved the occurrence of vegetable bodies in the dendritic variety of chalcedony named *mocha stone*, he must now admit that it does sometimes contain true vegetables, apparently of the nature of *conferva.* He observed these in specimens from Iceland and Catherinenburg. The same celebrated zoologist received from Dr. Lichtenstein, the traveller, a very remarkable agate, which was worn as a precious amulet by a Japanese prince. On examining it, he discovered in its interior the fructification of an unknown plant, somewhat resembling the *sparganium erectum.*

*Turquois.*—Dr. Langsdorf, who accompanied Captain Krusenstern in in his voyage round the world, presented to Blumenbach an uncut turquois, from Nischabar, in Eastern Persia. From the specimen it appears certain that this substance is not a petrifaction, but a particular mineral species which occurs in nests in beds of clay. According to the analysis of Dr. John, it appears to be nearly allied to mollite (siderite).

*Chromium in Chlorite.*—Mr. Bergweser Uttinger, of Sontkofen, has discovered from 1 to 1½ per cent. of chromium in a green-colored mineral heretofore considered as chlorite, and which gives a green color to some kinds of sandstone. This mineral occurs in masses, from the size of an inch to that of a pea, in grey shell limestone and grey sandy marl. It occurs also disseminated in compact and lenticular clay iron stone. It thus appears that chromium is far from being circumscribed in its distribution.

*Italian Rocks.*—Mr. Giuseppe Gautieri, principal inspector of forests in the kingdom of Italy, has lately examined the interesting valleys of Fiemme, Fassa, and Livinalunga, and finds the rocks to belong to the floetz trap formation, and to resemble, in every respect, the trap hills near Verona, Vicenza, and Padua, which have been described by many mineralogists as volcanic.

*Sarcocol.*—It has been generally believed that the substance called sarcocol is an exudation from the *penæa sarcocolla*; but there is very good reason to doubt the truth of the opinion. It was first taken up by Linnæus, and has been maintained ever since upon the authority of his name. Professor Thunberg has published an account of the genus penæa in the *Gesellschaft Naturfarschender freunde zu Berlin Magazin* for 1807, p. 121. The species are ten. They all grow at the Cape of Good Hope, and have been seen no where else. The penæa sarcocolla grows on the mountains of Hottentot Holland, and in those below the west side of the Table Mountain. Now Thunberg, who was on the spot, expressly affirms that sarcocol is neither collected nor known in that country.

It would have some tendency to throw light upon the plants that yield several resins, and gum resins, with the history of which we are at present unacquainted, if any wholesale druggist, or merchant, in London, who is in the habit of importing these articles, would state the countries from which they are brought.

*Freezing of Alcohol.*—Mr. Hutton, of Edinburgh, by a new method, which he has not thought proper to make known, but which he conceives capable of producing an unlimited degree of cold, has succeeded in freezing alcohol of the specific gravity of 0·798, and even of a specific about as low as 0·790 at the temperature of 60°. The alcohol, he conceives, froze at the temperature of — 110°; but no stress can be laid upon any such determination, as the contraction of the alcohol at the instant of freezing is probably irregular. It divided into three layers. The uppermost, yellow, consisted probably of the oils to which spirit owe their flavor, and which could not be removed by the concentration. The lowermost was the alcohol, probably still retaining water. This is nearly a tasteless liquid, according to Mr. Hutton: what the middle stratum is, he does not mention. This discovery of Mr. Hutton's is of moment, as it removes the only anomaly that existed against the general law, that all liquids become solid when exposed to a sufficient degree of cold.—*Annals of Philosophy*.

---

Since the publication of Mr. Adams's work on Diseases of the Eye, he has successfully performed the operation proposed in it for the cure of the complicated form of ectropium caused by cicatrices from small-pox, burns or scalds of the cheek and lid, &c. The operation was conducted as was described in page 315 of the last number of this Journal, and the result was precisely what he anticipated would happen. The extended raw surface produced by the separation of the lid from the cheek became covered by the production of new skin, excepting a small part near to the inner canthus, where it re-united. This was a second time separated, and a part of the conjunctiva on the inside of the lid, immediately opposite to it, removed with curved scissars; and, in order to prevent the integuments from again re-uniting before the conjunctiva had healed, the dissected part was repeatedly touched with argentum nitratum. At the end of a week, the contraction in the wound of the conjunctiva had drawn up this part of the lid in its natural situation (the outer part of the lid having been restored to its place by the first operation), when the external wound was suffered to cicatrize, and the original deformity can now be scarcely traced.

Mr. Astley Cooper saw this patient when cured, and, after comparing the lid with drawings which had been taken previously and subsequent to the operation for the purpose of publication, he, with his accustomed liberality, expressed his warm approbation at the successful result of this the first trial of a newly-invented operation for the cure of a disease which has hitherto been considered incurable by the most eminent writers on the subject.

---

Dr. Bateman has now in the press, A Practical Synopsis of Cutaneous Diseases according to the arrangement of Dr. Willan, exhibiting a concise view of the diognostic symptoms and the method of treatment. This is intended to contain an epitome of the whole of Willan's eight orders, four only of which have been completed and published. Its object is to present an outline of the system, and to detail briefly the characteristic appearances and progress of each disease, and the principles of treatment, without omitting altogether the historical and philosophical inquiries.

---

Mr. Johnson, Surgeon in Lancaster, has in the press an Essay on the Signs of Murder on New-born Children, principally translated from the French of De Mahord.

---

As a sign of the increasing progress of Medical Science in this country, it gives us pleasure to announce the institution of a society at Plymouth Dock, under the title of The Western Medical and Chirurgical Society. Its express objects are the diffusion of the various discoveries and improvements in the science of medicine, and the general advancement of medical literature.

Dr. Reid

Dr. Reid will commence his next Course of Lectures on the Theory and Practice of Medicine, on Monday the 17th of May, at ten o'clock in the morning, at his house in Grenville-street, Brunswick Square.

---

Dr. Adams will commence his Course of Lectures on the Institutes and Practice of Medicine, early in June.

---

*Theatre of Anatomy, Greville-street, Hatton-Garden.*—Mr. Taunton's Summer Course of Lectures on Anatomy, Physiology, Pathology, and Surgery will commence on Saturday, May the 22d, 1813, at eight o'clock in the evening, precisely, and be continued every Tuesday, Thursday, and Saturday, at the same hour.

---

Mr. Chevalier will commence a Summer Course of Lectures on the Principles and Practice of Surgery, on Monday the 16th of May, at eight o'clock in the morning, at his house, No. 20, South Audley-Street, Grosvenor Square.

---

Dr. Clutterbuck will begin his Summer Course of Lectures on the Theory and Practice of Physic, Materia Medica, and Chemistry, the first week in June, at his house, No. 1, in the Crescent, New Bridge-Street, where they will be continued daily, at ten o'clock in the morning. Two distinct courses are given on the alternate days, viz. on the Theory and Practice of Physic, on Mondays, Wednesdays, and Fridays; and on the Materia Medica, and Chemistry, on Tuesdays, Thursdays, and Saturdays.

